# ROS-Eliminating Carboxymethyl Chitosan Hydrogel to Enhance Burn Wound-Healing Efficacy

**DOI:** 10.3389/fphar.2021.679580

**Published:** 2021-06-14

**Authors:** Cheng Yang, Yuhui Chen, Hai Huang, Shicai Fan, Chengliang Yang, Liping Wang, Wenqiang Li, Wenxin Niu, Jianwen Liao

**Affiliations:** ^1^ Center for Orthopaedic Surgery, Department of Orthopaedic Trauma, The Third Affiliated Hospital of Southern Medical University, Guangzhou, China; ^2^ Department of Orthopedics, Affiliated Hospital of Youjiang Medical University for Nationalities, Baise, China; ^3^ UniSA Clinical & Health Science, UniSA Cancer Research Institute, University of South Australia, Adelaide, SA, Australia; ^4^ Gungdong Provincial Engineering Technology Research Center for Sports Assistive Devices, Guangzhou Sport University, Guangzhou, China; ^5^ Yangzhi Rehabilitation Hospital, Tongji University School of Medicine, Shanghai, China

**Keywords:** ROS-sensitive, carboxymethyl chitosan, hydrogel, macrophages, wound healing

## Abstract

Overexpression of reactive oxygen species (ROS) can lead to chronic inflammation, which limits skin wound healing. Therefore, it is of great significance to develop materials that can locally control the adverse reactions caused by excessive ROS. In this research, an ROS-sensitive hydrogel with strong free radical scavenging ability was prepared by introducing the thione (Tk) group into carboxymethyl chitosan (CMCTS) hydrogel. CMCTS hydrogel was cross-linked by NH_2_-Tk-NH_2_ agent and loaded curcumin (Cur), which possessed favorable nontoxicity, water absorption, mechanical property, biodegradability, drug release behavior, the M2 phenotype, and inflammatory factor regulating the capacity of macrophages. It is worth noting that Cur@CMCTS-Tk hydrogel can significantly inhibit oxidative damage of human fibroblasts in the H_2_O_2_-induced microenvironment and protect their viability by reducing the production of intracellular ROS. *In vivo*, ROS-removing hydrogel effectively accelerated the process of wound healing and possessed good regenerative properties, including hair follicle formation, promotion of new blood vessel formation, and highly orderly arrangement of collagen fibers in the full-thickness skin burn defect rat model. Hence, we expect that the Cur@CMCTS-Tk hydrogel could be used for wound treatment and tissue regeneration due to the ability to scavenge excess ROS.

## Introduction

Reactive oxygen species (ROS), which play vital roles in the normal metabolism and pathological process of humans, are signaling molecules, including superoxide radical (O_2_-), hydrogen peroxide (H_2_O_2_), and hydroxyl radical (-OH) (Privat-Maldonado et al., 2019; Yao et al., 2019). Excessive ROS production, however, can induce harmful processes, such as inflammation, necrosis, and cicatrization, to delay the healing of skin wounds and regeneration of damaged tissue (Mittal et al., 2014; Blaser et al., 2016). Therefore, designing a novel biomaterial that can locally control the excess ROS impairing cutaneous wound recovery and accelerate the regeneration process is urgently needed (Thannickal and Fanburg, 2000; Dröge, 2002; Kietzmann, 2010).

Among the many biomaterials, hydrogels were applied to deliver ROS scavengers to targeted sites under controlled therapeutic doses. Considering hydrogels can be used as a sustainable host of ROS scavengers, here is a growing hotspot that hydrogels as ROS-modulating materials are available for a variety of biomedical applications, for example, wound healing and tissue regeneration (Hu et al., 2020; Martin et al., 2020; Thi et al., 2020; Zhao et al., 2020).

As a special derivative of chitosan, carboxymethyl chitosan (CMCTS) was synthesized by replacing either or both of the amino (NH_2_) and hydroxyl (OH) functional groups in the glucosamine units with carboxymethyl (-CH_2_COOH) substituents (Xu et al., 2021; Zhang et al., 2021). CMCTS possesses high viscosity, low toxicity, and favorable biocompatibility, and all these excellent physical, chemical, and biological properties make this derivative become one of the research focuses in recent years (Fonseca-Santos and Chorilli, 2017; Shariatinia, 2018). Besides, CMCTS also have a unique advantage in forming hydrogel because the presence of carboxyl groups allows CMCTS to be constructed into materials *via* chemical cross-linking methods. It is well-known that curcumin (Cur) extracted from the rhizome of turmeric is one kind of natural polyphenols and has anti-inflammatory and antioxidant properties. In recent studies, curcumin was reported to not only scavenge excess ROS but also reduce cellular expression of pro-inflammatory cytokines (IL-6 and TNF-α) (Kasiewicz and Whitehead, 2016; Barchitta et al., 2019; Liczbiński et al., 2020; Vallée and Lecarpentier, 2020).

**SCHEME 1:**
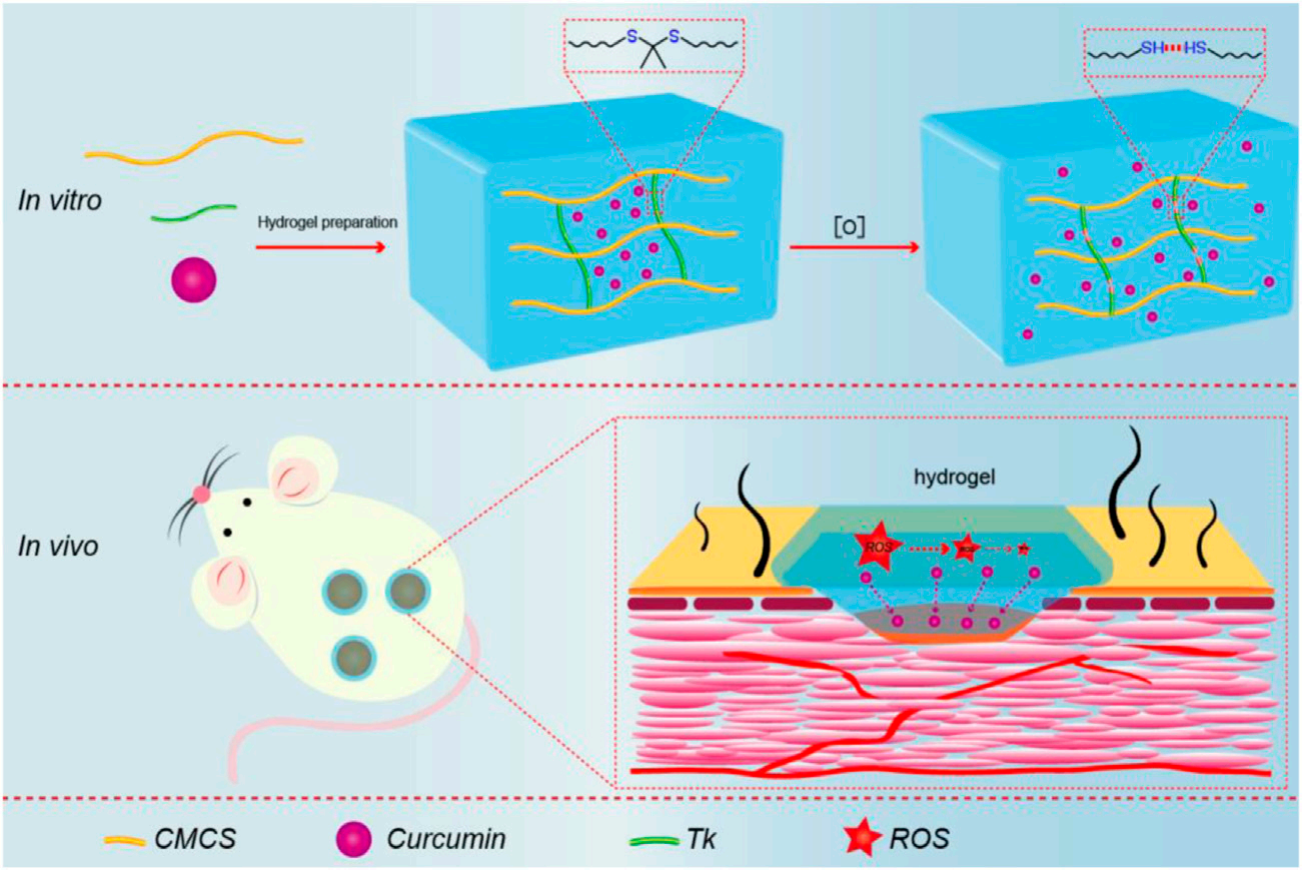
Schematic illustration of synthesis procedure for Cur@CMCTS-Tk and the accelerating release of Cur in the burn wound rat model.

Therefore, CMCTS-based hydrogel cross-linked by an ROS-sensitive linker and loaded with curcumin (Cur) was prepared to promote burn wound healing. In the microenvironment of the burn wound, such a hydrogel can clear superfluous ROS due to the presence of ROS-sensitive cross-linkers. In the meantime, with the degradation of the hydrogel, the loaded Cur was released from the interior of the hydrogel to further sweep away ROS and inhibit inflammation. After the preparation, we assessed the morphology, FTIR, Cur delivery property, water absorption, water vapor transmission, mechanical property, cytotoxicity, and macrophages phenotype *in vitro*. Subsequently, the hydrogel in full-thickness skin burn defect rats was applied to investigate the recovery efficiency, inflammatory factor expression, neovascularization, and collagen fiber alignment in the wound areas. As displayed in Scheme 1, we expect that the Cur@CMCTS-Tk hydrogel could be used for wound treatment and tissue regeneration due to the ability to scavenge excess ROS.

## Materials and Methods

### Materials

Carboxymethyl chitosan ((CMCTS, viscosity 1,000 mPs), substituted ratio >90%) was purchased from Dalian GlycoBio Co., Ltd (China), 2,2′-(propane-2,2-diyldisulfanediyl) diethanamine (NH_2_-Tk-NH_2_, Tk) was purchased from Cassim (Xi’an) Biotechnology Co., Ltd. (China), and enhanced green fluorescent protein (EGFP) plasmid was purchased from Hanbio (Shanghai) Co., Ltd. (China). The following materials were all obtained from Aladdin: H_2_O_2_ (30 wt% in H_2_O), Cur, 1-ethyl-3-(3-dimethylaminopropyl)-carbodiimide (EDC), N-hydroxysuccinimide (NHS), and lipopolysaccharide (LPS). All drugs and reagents were of analytical grades so that no additional refinement was required.

### Synthesis of Cur@CMCTS-Tk Hydrogels

EDC/NHS–mediated reaction was used to synthesize CMCTS-Tk polymers especially, and amide coupling reaction happened between the carboxylic groups (CMCTS) and the amino groups (NH_2_-Tk-NH_2_). In brief, first, 5 g of CMCTS was dissolved in 150 ml of distilled water at 40°C. Simultaneously, 2 g NH_2_-Tk-NH_2_ was dissolved in 125 ml buffer solution and 2 g EDC (10 mmol) and 1 g NHS (13.9 mmol) was also added to activate the carboxylic groups of CMCTS. Subsequently, the pH value of the solution stayed at 6.0 after stirring for 1 h. The purification of CMCTS-Tk was divided into two steps. The first step is to remove excess EDC/NHS, the product is placed into a dialysis bag with a molecular cutoff point of 3,500 Da for 3 days to remove the excess EDC and NHS. The second step is to remove the excess or unreacted Tk, the product is placed into a vessel filled with anhydrous acetone under a shaker (200 rpm) for 24 h. The product is washed with anhydrous acetone thrice and with deionized water once. Then 100 mg Cur was added to 10 ml ethyl alcohol solution and then CMCTS-Tk hydrogel (5 g) was placed in a shaker (100 rpm/s) overnight at 40^°^C to fully loading 48 h. After loading Cur, the excess unloading Cur is taken to measure the concentration for calculating the loading quality into CMCTS-Tk hydrogel *via* the UV-Vis method. Finally, the Cur@CMCTS-Tk hydrogel was obtained following the process of filtration and lyophilization. In addition, a control group of hydrogel (named Cur@CMCTS) was prepared in which Tk-c [NH_2_-(CH_2_)_6_-NH_2_] was used to replace NH_2_-Tk-NH_2_, and the other steps remained unchanged.

### Morphology and FTIR of Hydrogels

Morphology: The final hydrogels were cut and coated with gold by sputtering, and a field emission scanning electron microscope (SEM) (Philips LEO1530 VPSEM) was used to observe the cross section morphology of the hydrogel.

FTIR analysis: The cross-linked reaction between NH_2_-Tk-NH_2_ and CMCTS molecules was identified through an FTIR spectrophotometer (Bruker Optics Inc.). For the measurement, KBr was introduced to the sample to form transparent pallets. The test was conducted at room temperature with wavenumber ranges from 4,000 to 400 cm−^1^ and a resolution of 4 cm−^1^.

^1^H NMR analysis: The purity of CMCTS-Tk hydrogel, CMCTS, and Tk were determined by the proton nuclear magnetic resonance (^1^H NMR) spectra.

### *In Vitro* Delivery of Cur by Hydrogels

To examine the release behavior of Cur under H_2_O_2_ condition, 2.0 g Cur@CMCTS and Cur@CMCTS-Tk hydrogel were placed into 10 ml of cell lysate (RIPA lysis buffer) medium at 37°C. After 5 h, the releasing medium was added into 50 mM H_2_O_2_. At the appointed time, we took 8 ml of the mixed solution to investigate the release ratio *via* the ultraviolet spectrophotometer and replaced it with the same volume of fresh buffer solution instead.

### Swelling Ratio of Hydrogels

In order to measure the swelling rate of CMCTS hydrogels, 200 mg Cur@CMCTs and Cur@CMCTS-Tk hydrogels were prepared with Tk-c and Tk cross-linking agents separately and then immersed in deionized water for 22 h after freeze-drying, respectively. After removing the excess water, the hydrogels were weighed (wet weight). Then the hydrogel sample was frozen and lyophilized to weigh again (dry weight), and the formula for determining the swelling ratio is given in Eq. 1(Augustine et al., 2021; Balakrishnan et al., 2005).

$$\text{Swelling}\;\text{Ratio}\left(\text{SR}\right)=\frac{W_{wet}}{W_{dry}}.$$ (1)

Equation 1. Swelling ratio determination for hydrogels.

### Water Vapor Transmission Rate and Mechanical Characterization of Cur@CMCTS-Tk Hydrogel

Based on the American Society for Testing Material (ASTM) standard E96-00 (Queen et al., 1987), the moisture permeability of Cur@CMCTS-Tk hydrogel was determined by water vapor transmittance (WVTR). Briefly, hydrogels were placed over the mouth of a 40-mm-diameter cylindrical glass bottle containing deionized water, and the rim of the bottle is tightened to prevent water vapor from escaping from the edge. The bottle was placed in an environment of 37°C and 35% humidity for 24 h, while the relation curve between time and weightlessness was recorded and drew. WVTR was calculated by the following equation based on the slope of the curve.

$$WVTR=\frac{slope\times 24}{A}g/m^{2}/day.$$ (2)

Eq. 2. WVTR of hydrogels, A indicates the test area of hydrogels (m^2^).

Mechanical strength: Before the analysis of mechanical strength using a rheometer, the hydrogels were treated with different times of H_2_O_2_. Subsequently, storage modulus (G′) was measured through frequency (range from 0.1 to 10 Hz), strain sweeping (at a maximum strain of 10%), and oscillatory (with a frequency of 1.0 and 100 Hz) mode.

### Cytotoxicity of Hydrogels

The biocompatibility of hydrogels was studied using the fibroblast (L929) cells. First of all, the L929 cells were cultured with the DMEM media containing 10% fetal bovine serum (FBS), penicillin (100 UmL^−1^), and streptomycin (100 UmL^−1^) under appropriate humidified incubating conditions (at 37 C in 5% CO_2_). Then the cells were transfected by EGFP plasmid to emit green fluorescence for a laser scanning confocal microscope (LSCM, 510Meta Duo Scan, Zeiss, Germany) observation. After 7 days of culture, the cells were separated *via* trypsinization and centrifugation processes. For the biocompatibility analysis, the CMCTS-Tk and Cur@CMCTS-Tk hydrogels were sterilized by Co-60 for 10 kGy. Finally, the L929 cells were seeded in 5 × 10^4^ cells per well of a 24-well plate, and the LPS was also added to the culture medium to simulate the ROS environment *in vitro*.

LSCM fluorescence imaging: It was used to visualize the L929 cells after culturing with different hydrogels. The cells were captured using a laser scanning confocal microscope (LSCM, 510Meta Duo Scan, Zeiss, Germany) with the EGFP excitation wavelength 486 nm and emission wavelength 509 nm at the 7 days.

Cells migration: The effect of hydrogel on the migration property of L929 cells was assessed by *in vitro* wound healing migration experiment. Hydrogels were placed at the bottom of the 24-well plate and then L929 cells were inoculated on it. After 24 h, cell scratches were formed with the tip of the sterile pipette. Cultured for 24 h later, the cells were fixed with 4% paraformaldehyde at room temperature for 15 min and DAPI staining and microscope photography were performed.

MTT assessment: The cell cytotoxicity was tested by the MTT method, and the cell survival percentage was defined as OD_exp_/OD_con_. OD_exp_ represented the optical density in the experimental group, while OD_con_ was for the control group.

### Effects of Hydrogels on RAW 264.7 Cells Polarization, Inflammatory Response, and Cytokine Expression

To analyze the effects of Cur@CMCTS-Tk hydrogel on macrophages phenotype switch and inflammatory response, a flow cytometer (FCM) and a Western blot analysis were applied. To stimulate the ROS microenvironment, LPS (100 ng/ml) was chosen and co-cultured with Cur@CMCTS-Tk hydrogel. At last, 1.0 × 10^5^ RAW 264.7 cells were cultured with CMCTS-Tk or Cur@CMCTS-Tk hydrogel and LPS condition.

Western blot analysis: Fibroblasts were rinsed in phosphate buffer saline (PBS) and mixed with a radioimmunoprecipitation assay (RIPA) buffer containing 1% (v/v) phenylmethylsulfonyl fluoride (PMSF). The protein was electrophoretically resolved (120 V) on a 12% SDS-polyacrylamide gel and transferred (350 V) to PVDF membrane for 90 min and incubated with 5% skim milk. Afterward, the PVDF membrane was incubated overnight with primary antibodies at 4°C and washed with TBST thrice, for 10 min each time. Next, the membrane was blotted with peroxidase-conjugated secondary antibodies and washed the same number of times as in the previous step with TBST. Visualization of proteins was performed by the chemiluminescent signal following the instructions of the manufacturer. Primary antibodies for TNF-α (ab255275) and IL-10 (ab189392) monoclonal antibodies were used.

FCM analysis: After incubation for 48 h, 10% mouse serum was used to block the RAW 264.7 cells for 30 min. The cells were then incubated in the mixed solution combining rabbit CD86 (ab242142) and CD206 (ab223961) monoclonal antibody (dissolved in PBS) with 0.05% proclin300 and 1% BSA for at 4°C 30 min. After washing with PBS thrice, the RAW 264.7 cells were placed in PBS and analyzed by flow cytometry (Beckman Coulter, California, United States). Besides, the inflammation-associated protein of TNF-α and IL-10 was analyzed by using a flow cytometer which is incubated with rabbit TNF-α (ab255275) and IL-10 (ab215975) monoclonal antibody.

### *In Vivo* Wound Repair

Based on previous studies (Lin et al., 2020), the effect of hydrogels on wound healing *in vivo* was evaluated in a full-thickness burn rat model for up to 21 days. Animal experiments were performed according to the approval of the Animal Ethics Committee of Jinan University, in accordance with relevant laws and institutional guidelines. Specifically, 36 male Sprague Dawley (SD) rats (2–3 months of age) weighing 250 g were intraperitoneally injected with ketamine and thiazide at 40 and 5 mg/kg, respectively. After shaving the operative dorsal skin region of rats and disinfecting with 75% ethanol, a scalding machine was applied to burn for 10 s at 95 ± 1 C. A full-thickness circular wound was created about 2 cm in diameter by using forceps and scissors to remove the damaged tissue. PBS, CMCTS, Cur@CMCTS, CMCTS-Tk, and Cur@CMCTS-Tk hydrogels were placed on the wound fixed with an elastic bandage to promote healing. All rats were kept alone in cages and fed with enough food and water till they were sacrificed. At regular intervals, the wound appearance was photographed *via* camera and the wound trace was plotted by Adobe Illustrator (AI) software. According to the wound area at different times (days 0, 7, 14, and 21), the percentage of wound contraction was calculated using Eq. 3.

$$Wound\;contraction=\frac{Area_{d0}-Area_{dn}}{Area_{d0}}\times 100\%.$$ (3)

Eq. 3. The percentage of wound contraction, where d0 is on day 0, and dn is on days 7, 14, and 21, respectively.

### Histological and Immunohistochemical Staining

The rats were sacrificed on day 21 after skin burns, and the wound with the surrounding skin was excised for histological detection. Skin tissues were fixed in formaldehyde for 24 h, dehydrated in an ethanol solution, and embedded in paraffin waxes. Histological sections were cut as 4.5 μm and stained with hematoxylin and eosin (H&E) and Masson staining for histological analysis.

The expression levels of TNF-α and CD31 were detected by immunohistochemistry. The slides were incubated with the primary antibody at 4°C overnight and with a secondary antibody at room temperature for 90 min. Photomicrographs were observed under a light microscope (DS-Fi3; Nikon, Japan).

### Statistical Analysis

Data were evaluated using GraphPad Prism 6 software followed by the Student’s unpaired *t*-test. We define *p* < 0.05 as statistically significant.

## Results and Discussion

### Morphologies, Compositions, and Delivery Property of Hydrogels

**FIGURE 1:**
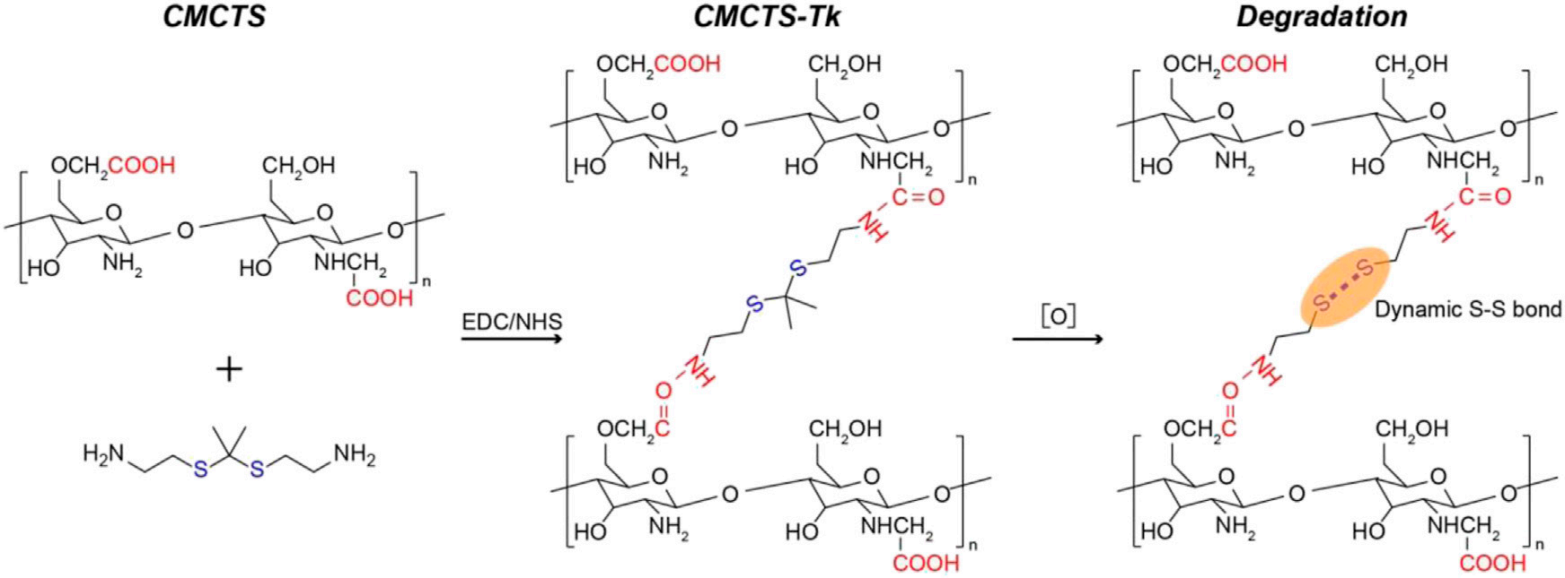
Schematic representation of Cur@CMCTS hydrogel preparation and degradation procedures.

Cur@CMCTS-Tk and Cur@CMCTS hydrogels were synthesized *via* CMCTS monomers in an aqueous phase system by using Tk and Tk-c as the cross-linker, EDC/NHS as the activating agent. The Cur@CMCTS hydrogel cross-linked and degradation procedures were shown in Figure 1.

In Supplementary Figure S1, the ^1^H NMR spectrum of CMCTS, Tk, and CMCTS-Tk was shown. For CMCTS ^1^H NMR, the peaks shows broader. The resonance of acetyl (-C(O)CH3) protons can be found at the chemical shift 2.0 ppm. The methylene proton in O- and N-substituted carboxymethyl chitosan appeared at the shift 3.91 and 3.31 ppm, respectively. The shift in the range of 3.5–4.0 ppm is corresponding to the proton from carbon atom C3–C6 from glucopyranose unit. For the Tk ^1^H NMR, the peaks appear at 2.81, 2.64, 1.51, and 1.22 ppm, which are kept in accordance with the previous study (Li et al., 2020). For the ^1^H NMR of CMCTS-Tk, the curve kept similarly with the CMCTS. In addition, the Tk characteristic peak appeared, indicating the successfully cross-linked between CMCTS and Tk.

**FIGURE 2:**
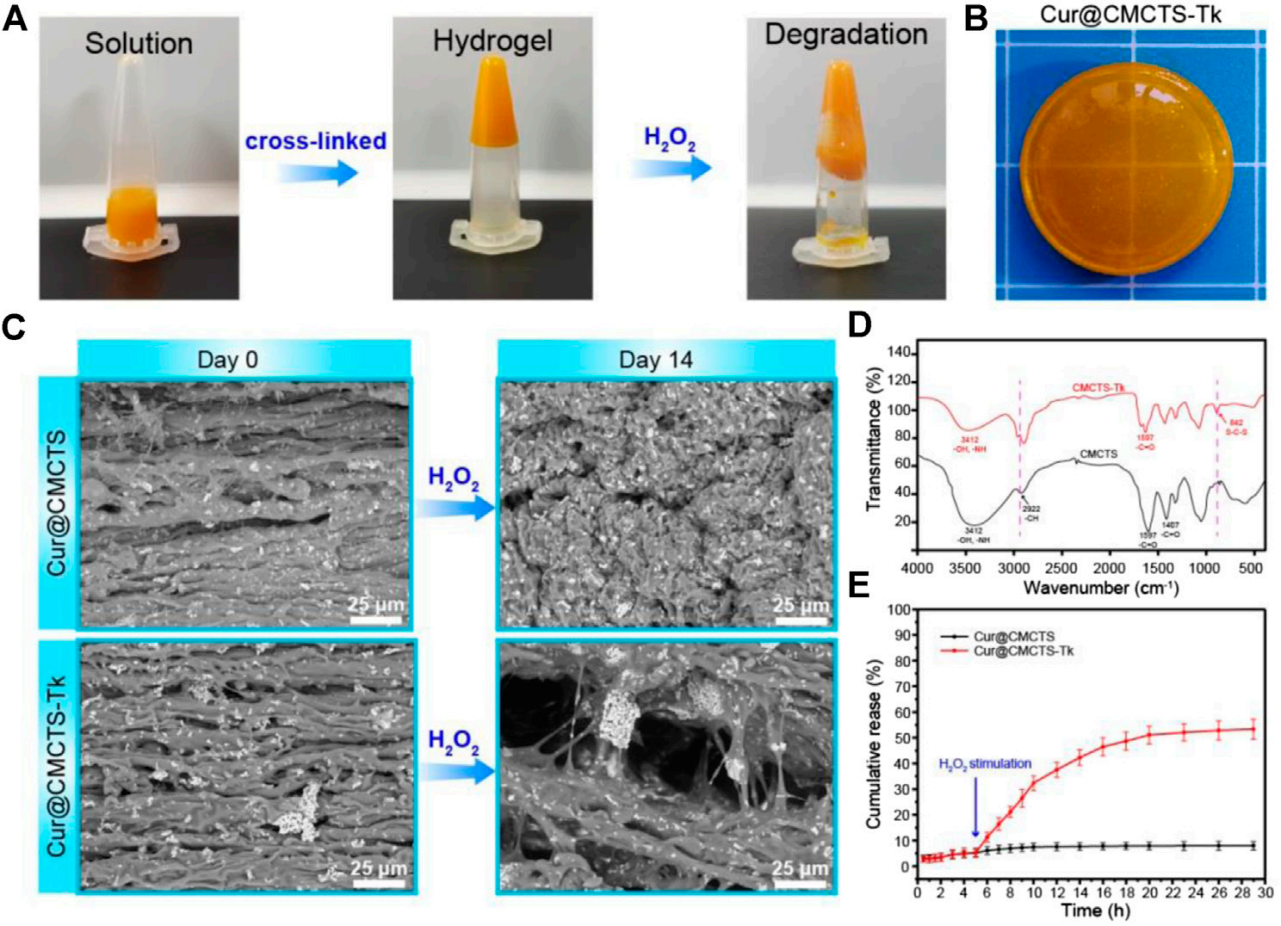
**(A)** Sol−gel transition behavior of Cur@CMCTS-Tk hydrogel. **(B)** Image of Cur@CMCTS-Tk hydrogel for burn wound repair. **(C)** SEM image of Cur@CMCTS and Cur@CMCTS-Tk hydrogels treated with H_2_O_2_. **(D)** FTIR analysis of CMCTS and CMCTS-Tk hydrogels. **(E)** Cur delivery curve from Cur@CMCTS and Cur@CMCTS-Tk hydrogels treated with H_2_O_2_ at 5 h.

In Figure 2A, the results of the tube inversion test indicated Cur@CMCTS-Tk hydrogel showed good gelation within the appropriate reaction time (Kim et al., 2016). When Cur@CMCTS-Tk hydrogel was treated with H_2_O_2_ for 7 days, the hydrogel became degradation indicating the ROS sensibility ability. The appearance of Cur@CMCTS-Tk hydrogel (Cur/Cur@CMCTS-Tk is 1.76 wt%) developed for rats full-thickness burn repair is shown in Figure 2B.

The cross-section morphology of Cur@CMCTS and Cur@CMCTS-Tk hydrogels treated with H_2_O_2_ were observed by SEM (Figure 2C). Both Cur@CMCTS and Cur@CMCTS-Tk hydrogel possessed a relatively tight structure with a limited micro-pore that favored the micromolecule gas permeation but did not favor Cur delivery. Notably, obvious Cur residues were seen on the hydrogel surface, indicating the complete dispersion inside hydrogel. After being treated with H_2_O_2_, the pore diameter became larger for the cross-linked structure degradation.

As depicted in Figure 2D, the FTIR spectra of CMCTS-Tk and CMCTS were clearly displayed. CMCTS showed strong peaks at 1,597 cm−^1^ and 1,407 cm−^1^, which corresponded to the carboxy group and carboxymethyl group, respectively (Kalaithong et al., 2021). Besides, a wideband at 3,412 cm−^1^ meant stretching vibrations of O–H and N–H bonds, while 2,922 cm−^1^ for C–H bonds. After being cross-linked by NH_2_-Tk-NH_2_, CMCTS-Tk presented the enhanced characteristic absorption bands of methylene stretching peak at 2,890 cm−^1^ and a new stretching peak of S-C-S at 845 cm−^1^, indicating that NH_2_-Tk-NH_2_ was successfully grafted onto the CMCTS.

The H_2_O_2_-sensitivity delivery curve of Cur from Cur@CMCTS and Cur@CMCTS-Tk hydrogel is exhibited in Figure 2E. For Cur delivery in Cur@CMCTS-Tk hydrogel, a fast release of 53.4 ± 3.9% was observed after 5 h because the thioketal chain was broken by H_2_O_2_ attack, while almost no delivery was observed in Cur@CMCTS hydrogel due to the close integration of Cur and CMCTS chain (Shim and Xia, 2013; Pu et al., 2014). This Cur release feature is favorable for applying in ROS redundant wound. For the ROS-sensitivity hydrogel, if the burn wound possessed abundant ROS, the ROS-sensitivity hydrogel molecular chain will soon break and release curcumin to absorb the excess ROS. Once the ROS is controlled in a low level, the hydrogel molecular chain will stop the breakage and the curcumin will keep a stable release rate. The results demonstrated that Cur@CMCTS-Tk hydrogel could deliver continuously Cur under H_2_O_2_ condition. It have been demonstrated that when the curcumin concentration is more than 2.5 μg/ml, it will reduce the inflammatory factor production in LPS-induced macrophages (Ternullo et al., 2019). In this study, those continuously released curcumin concentrations are enough for cellular uptake. It can be supposed that curcumin cellular uptake is time dependent and occurs through a concentration gradient mechanism *via* membrane partitioning (Shefa et al., 2020).

### The Water Absorption and Water Vapor Transmission Rate of Hydrogels

**FIGURE 3:**
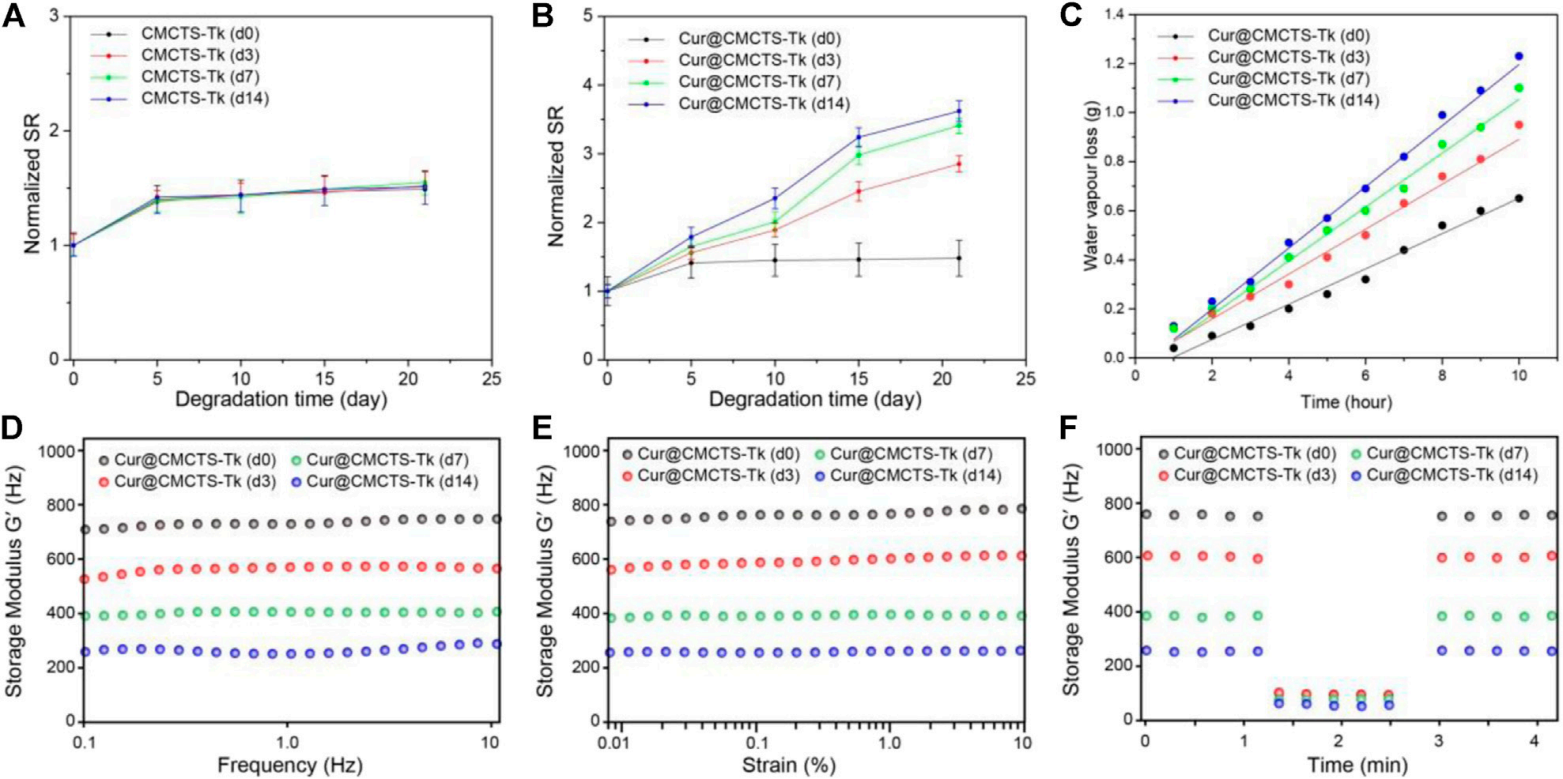
Normalized swelling ratio of CMCTS-Tk **(A)** and Cur@CMCTS-Tk **(B)** hydrogels treated with H_2_O_2_ for 0, 3, 7, and 14 days. **(C)** Water vapor loss of Cur@CMCTS-Tk hydrogel treated with H_2_O_2_ for 0, 3, 7, and 14 days. **(D)** Frequency-dependent (from 0.1 to 10 Hz), **(E)** strain sweeping (at a maximum strain 10%), and **(F)** oscillatory mode with a frequency (1.0 and 100 Hz) of storage modulus (G′).

The results of the water absorption test indicated that the water equilibrium swelling rate of Cur@CMCTS and Cur@CMCTS-Tk hydrogels in PBS solution was ∼48% (Figures 3A,B). Such a high fluid absorption capacity was essential for absorbing wound exudate and edema fluid. However, after being treated with H_2_O_2_, Cur@CMCTS-Tk hydrogel was significantly more swollen than the PBS-treated hydrogel, showing up as the bigger aperture.

The ideal wound dressing also could keep the wound moist by controlling water loss at the optimal rate. The evaporation rate of water from wound evaporation ranged from 2000 to 2,500 g/m^2^/day provides sufficient water to get rid of the risk of wound dehydration. Based on the slope of the chart (Figure 3C), the WVTR of Cur@CMCTS-Tk hydrogel treated with H_2_O_2_ for 0, 3, 7, and 14 days were ∼1,376, ∼1749, ∼2098, and ∼2,381 g/m^2^/day, respectively. The WVTR of Cur@CMCTS-Tk treated with H_2_O_2_ possessed an appropriate WVTR treated with H_2_O_2_ for 3 days, which is beneficial to maintain appropriate liquid balance on the surface of the wound.

### Rheology Characterization of Cur@CMCTS-Tk Hydrogels

To evaluate the degradation property of Cur@CMCTS-Tk hydrogels *in vitro*, the hydrogel was incubated with different concentrations of H_2_O_2_ and characterized by rheological property. As shown in Figures 3D–F, the rheological analysis suggested that the storage modulus (G′) of Cur@CMCTS-Tk hydrogel was irrelevant to frequency, which confirmed its hydrogel properties. In addition, the G′ of Cur@CMCTS-Tk hydrogel was time-dependent when treated with H_2_O_2_ and G′ on day 0 was ∼2.69 times than on day 14 in the frequency mode. The result demonstrated that the fluid property of Cur@CMCTS-Tk hydrogel became better to fit the wound healing after being treated with H_2_O_2_.

On the other hand, the G′ frequency at high (100 Hz) and low (1.0 Hz) shear frequency were measured to assess the self-healing ability of Cur@CMCTS-Tk hydrogel. The sharp drops of G′ of Cur@CMCTS-Tk hydrogel at high frequency verified its shearing refinement performance, while the fast recovery of G′ at low frequency after high frequency indicated that G′ has good self-healing property due to the formation of dynamic "S–S" bond. The results showed that Cur@CMCTS-Tk hydrogel treated with H_2_O_2_ could significantly accelerate its degradation *in vitro* and improve rheological properties.

### Cytotoxicity of Hydrogels

**FIGURE 4:**
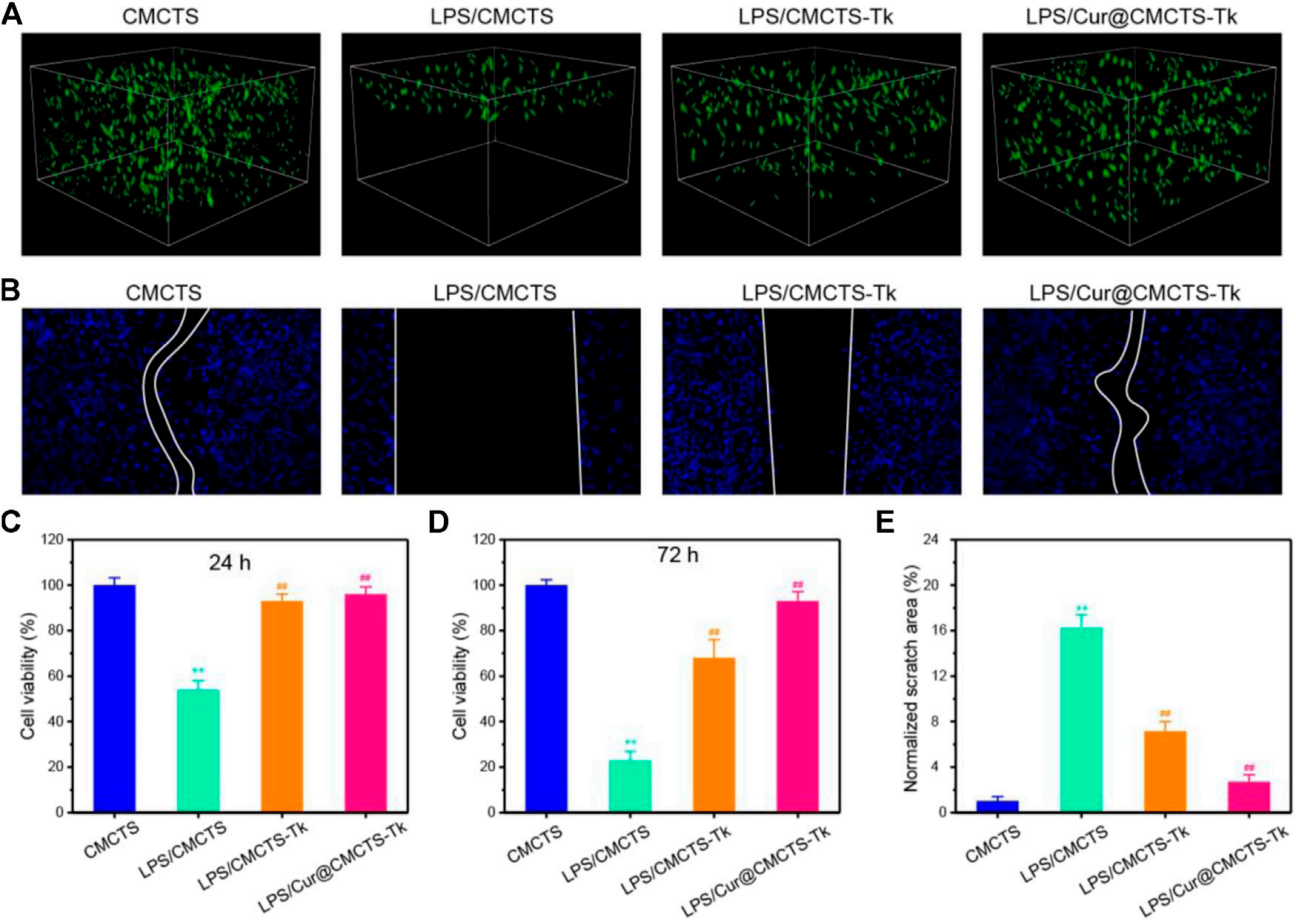
**(A)** The GFP-plasmid transfected cells were cultured with CMCTS (Control), LPS/CMCTS (LPS), LPS/CMCTS-Tk, and LPS/Cur@CMCTS-Tk hydrogel for 72 h. **(B)** Images for cell wound scratch assay of L929 cells at 72 h. **(C)** Cytotoxicity of Cur@CMCTS-Tk hydrogel to fibroblast cells by MTT assay at 24 and 72 h. **(D)** Quantitative analysis of the scratch area. The data were represented as mean ± SD (*n* = 6), **p < 0.01 vs. control; ^##^ *p* < 0.01 vs. LPS/CMCTS (LPS) group.

It should be biocompatible if a dressing material aims at repairing the wound. To explore the cytotoxicity of Cur@CMCTS-Tk hydrogel, the GFP plasmid–transfected fibroblast cells were implanted into Cur@CMCTS-Tk hydrogel surface (Figure 4A). For the CMCTS-Tk hydrogel group, a large number of fibroblast cells are proliferated into the hydrogel interior after culturing for 72 h. While for the LPS/CMCTS hydrogel group, the cells were only dispersed on the hydrogel superficial layer. For LPS/CMCTS-Tk and LPS/Cur@CMCTS-Tk group, the cells could continue to adhere to the hydrogel’s interior. The results may be due to the CMCTS-Tk hydrogel could eliminate ROS aroused by LPS and further promotes cell proliferation.

Here, the cell migration property was evaluated using the L929 cells (Figure 4B). The CMCTS-Tk and Cur@CMCTS-Tk hydrogels both could promote L929 cells migration when compared with the LPS groups (Figure 4E) because the CMCTS-Tk hydrogel could absorb redundant ROS brought by LPS. Besides, the released Cur also could eliminate cell inflammation, improve cell activity, and then promote cell proliferation and migration. The MTT assay was used to evaluate the toxicity of Cur@CMCTS-Tk hydrogel to fibroblast cells at 24 and 72 h (Figures 4C,D). Based on the international standard [ISO 10993-5:2009(E)], the cytotoxicity was divided into 0, 1, 2, 3, 4, and 5 grades, which correspond to the cell survival rate as 100%, 75 ∼ 99%, 50 ∼ 74%, 25 ∼ 49%, 1 ∼ 24%, and 0 grade, respectively. Among these six grades, grade 0 and grade 1 are considered as non-cytotoxic. When fibroblast cells are cultured with LPS/CMCTS hydrogel, the cytotoxicity is 54.1 ± 4.3 (24 h) and 22.7 ± 3.8 (72 h) corresponding to the 2nd grade and 3rd grade, which is harmful to cells. When cells were cultured with LPS/CMCTS-Tk or LPS/Cur@CMCTS-Tk hydrogel, the cytotoxicity was significantly decreased compared to that in the LPS/CMCTS group both on days 3 and 7. The cell survival percentage of these two hydrogels could both reduce the cytotoxicity brought by H_2_O_2_.

### Macrophage Phenotype Converted by Hydrogels

Macrophages are immune cells with a variety of functions and can be divided into M1 type and M2 type according to their activation state and function (Shen et al., 2020). M1 macrophages possess enhanced anti-inflammatory ability, secrete pro-inflammatory cytokines (such as TNF-α, IL-1, IL-6, and IL-23), and arouse ROS, while M2 macrophages can inhibit pro-inflammatory cytokines and secrete extracellular matrix components that may be necessary for the late stage of tissue repair (Chen et al., 2014). Granulation tissue formation, myofibroblast differentiation, matrix deposition, and angiogenesis also rely on the M2 phenotype (Murray et al., 2014).

**FIGURE 5:**
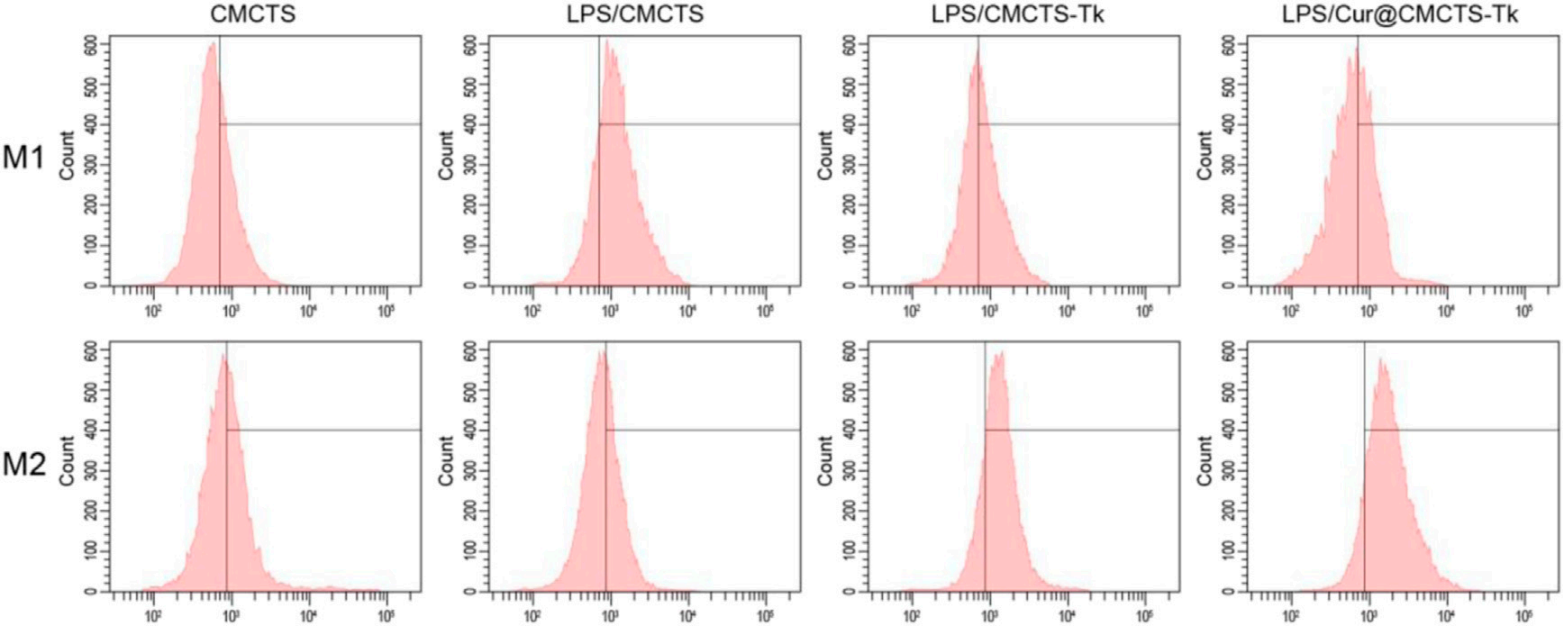
FCM results of RAW cells cultured with normal CMCTS hydrogel (control) and LPS/CMCTS (LPS), LPS/CMCTS-Tk, and LPS/Cur@CMCTS-Tk hydrogel after 24 h. RAW cells were stained with M1 marker CD86 and M2 marker CD206. The ratios of M1 and M2 phenotypes of macrophages were also presented.

To analyze the macrophage phenotypic switch influenced by Cur@CMCTS-Tk hydrogel, the M1 and M2 phenotypic markers of macrophages were detected. Macrophages expressing M1 marker CD86 and M2 marker CD206 were detected by FCM as shown in Figure 5. It can be seen that the RAW cells M2 phenotype was inhibited by LPS. While cultured with LPS/CMCTS-Tk and LPS/Cur@CMCTS-Tk hydrogel, the macrophages M2 were increased to 73.3 ± 4.8% and 83.4 ± 3.1%, respectively. On the other hand, RAW cells M1 phenotype was evoked by IPS when compared with the control group. While cultured with LPS/CMCTS-Tk and LPS/Cur@CMCTS-Tk hydrogel, the macrophages M1 were decreased to 42.1 ± 3.7% and 37.9 ± 4.5%, respectively. The results suggested that CMCTS-Tk and Cur@CMCTS-Tk hydrogels could reduce the inflammatory phenotype evoked by LPS. In addition, the detailed M1 and M2 phenotype statistical data were recorded in Table 1.

**TABLE 1:** The RAW cells M1 and M2 phenotype statistical data (mean ± SD, *n* = 3).

	CMCTS (%)	LPS/CMCTS (%)	LPS/CMCTS-Tk (%)	LPS/Cur@CMCTS-Tk (%)
M1	35.8 ± 3.4	73.1 ± 4.5	42.1 ± 3.7	37.9 ± 4.5
M2	43.1 ± 2.7	37.8 ± 3.6	73.3 ± 4.8	83.4 ± 3.1

### Effects of Hydrogels on Inflammation-Related Cytokine Expression in RAW Cells

**FIGURE 6:**
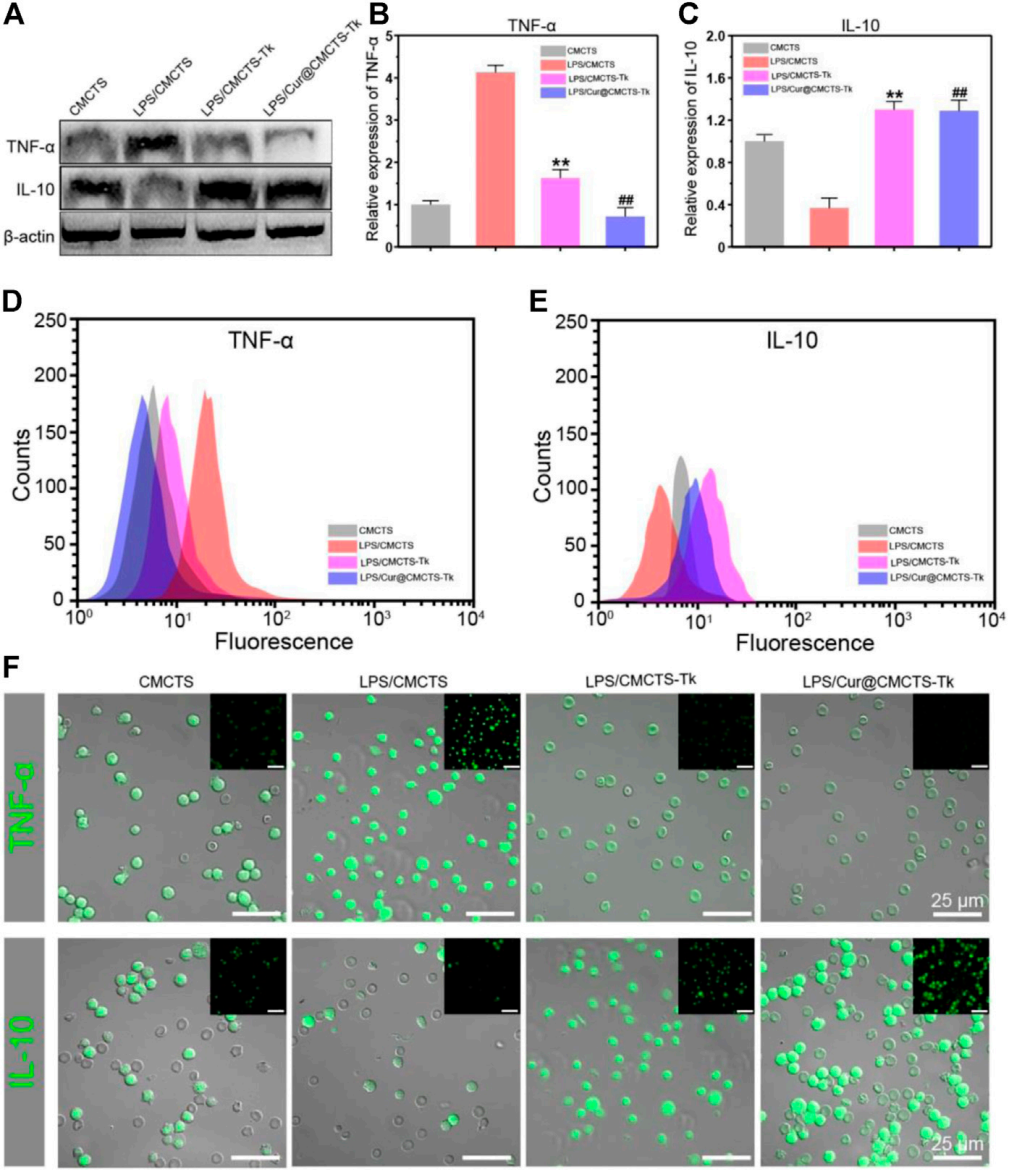
The protein expressions in RAW cells cultured with normal CMCTS hydrogel (control) and LPS/CMCTS (LPS), LPS/CMCTS-Tk, and LPS/Cur@CMCTS-Tk hydrogel after 24 h were analyzed using Western blot **(A)** and the TNF-α **(B)**, IL-10 **(C)** relative expression analysis. The FCM analysis **(D–E)** and immunofluorescence staining **(F)** of TNF-α and IL-10 in RAW cells. The data were represented as mean ± SD (*n* = 3). **p < 0.01 vs. control; ^##^ *p* < 0.01 vs. LPS group. The scale bar is 25 μm.

To investigate the inflammation-related cytokine expression in RAW cells after cultured with Cur@CMCTS-Tk hydrogel, RAW cells were cultured on Cur@CMCTS-Tk hydrogel with LPS and then analyzed by Western blot, FCM, and immunofluorescence method. Figure 6A shows the Western blot results that Cur@CMCTS-Tk hydrogel could activate the macrophages to boost the anti-inflammatory factors IL-10 expressions (Figure 6C) and inhibit the pro-inflammatory factors TNF-α expression (Figure 6B) under LPS environment. In order to evaluate cytokine levels quantitatively, the FCM was applied to measure the expression of TNF-α and IL-10 in RAW cells (Figures 6D,E). The FCM result was consistent with the Western blot result, guaranteeing that CMCTS-Tk and Cur@CMCTS-Tk hydrogel could suppress the secretion of inflammatory cytokines TNF-α and promote anti-inflammatory cytokines IL-10 expression. Finally, to more visually reflect the expression of TNF-α and IL-10, their antibodies were used to measure RAW cells using immunofluorescence staining. It can be seen from Figure 6F that Cur@CMCTS-Tk hydrogel reduced the expression of TNF-α but improved IL-10 in macrophages. All the above results could co-prove that CMCTS-Tk and Cur@CMCTS-Tk hydrogel inhibited the expression of the inflammatory cytokines and enhanced anti-inflammatory cytokines expression under the LPS environment.

### *In Vivo* Wound Repair

**FIGURE 7:**
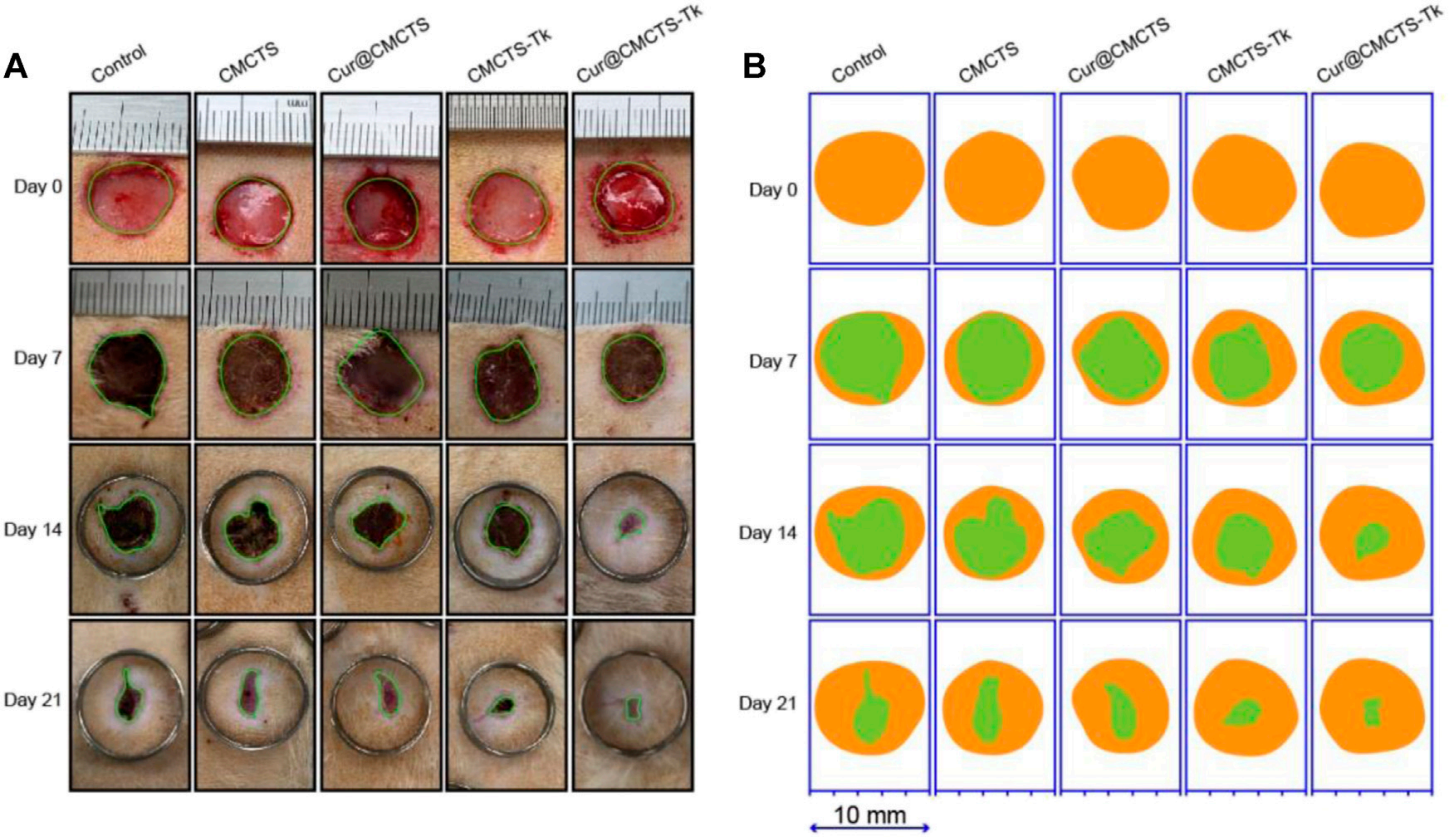
**(A)** Digital photos of wounds on the dorsum of rats after treatment by Cur@CMCTS-Tk hydrogel for up to 21 days using no treatment, CMCTS, Cur@CMCTS, and CMCTS-Tk hydrogel as the controls. In the early seven days, the wound was rewetted at an interval of 8 h by two to three drops of PBS (37°C), allowing Cur delivery; **(B)** wound trace was drawn by Adobe Illustrator software. Yellow: 0 d, green: real-time day. The data were represented as mean ± SD (*n* = 3) (*p < 0.05, **p < 0.01, ***p < 0.001).

CMCTs, Cur@CMCTS, and CMCTS-Tk hydrogels were used as the control groups to evaluate the wound healing effect of Cur@CMCTS-Tk hydrogel *in vivo*. Figure 7A illustrates the progress of wound closure after hydrogels treatment and the wound trace drawn by AI software was presented in Figure 7B. During the early 7 days, rewetting all hydrogels with two to three drops of PBS every 8 h to keep the wound moist. On the 7th day, the wound surface of each part was significantly reduced when some suppuration appeared, indicating the wound became inflamed. On day 14, all wounds of the control groups contracted into irregular circles with a contraction range of 56–60% and red granulation tissue was formed. At the same time, the scab of the Cur@CMCTS-Tk hydrogel group basically disappeared, and the wound recovery efficiency was about 83%, which was significantly better than the control groups. Here, this accelerated repair efficiency could be explained in two steps. For the first step, Cur@CMCTS-Tk hydrogel was attacked by excrescent ROS, the redundant ROS was absorbed by Cur@CMCTS-Tk hydrogel when the thioketal group is broken. Second, as the Cur@CMCTS-Tk hydrogel degraded, its fluidity enhanced, and then Cur released from the Cur@CMCTS-Tk hydrogel interior into the wound defect area. These results indicated that Cur@CMCTS-Tk hydrogels exhibited the most significant healing effect in all groups due to the gradual transmission of the elimination of Cur and ROS during wound healing.

### H&E and Masson’s Trichrome Staining

**FIGURE 8:**
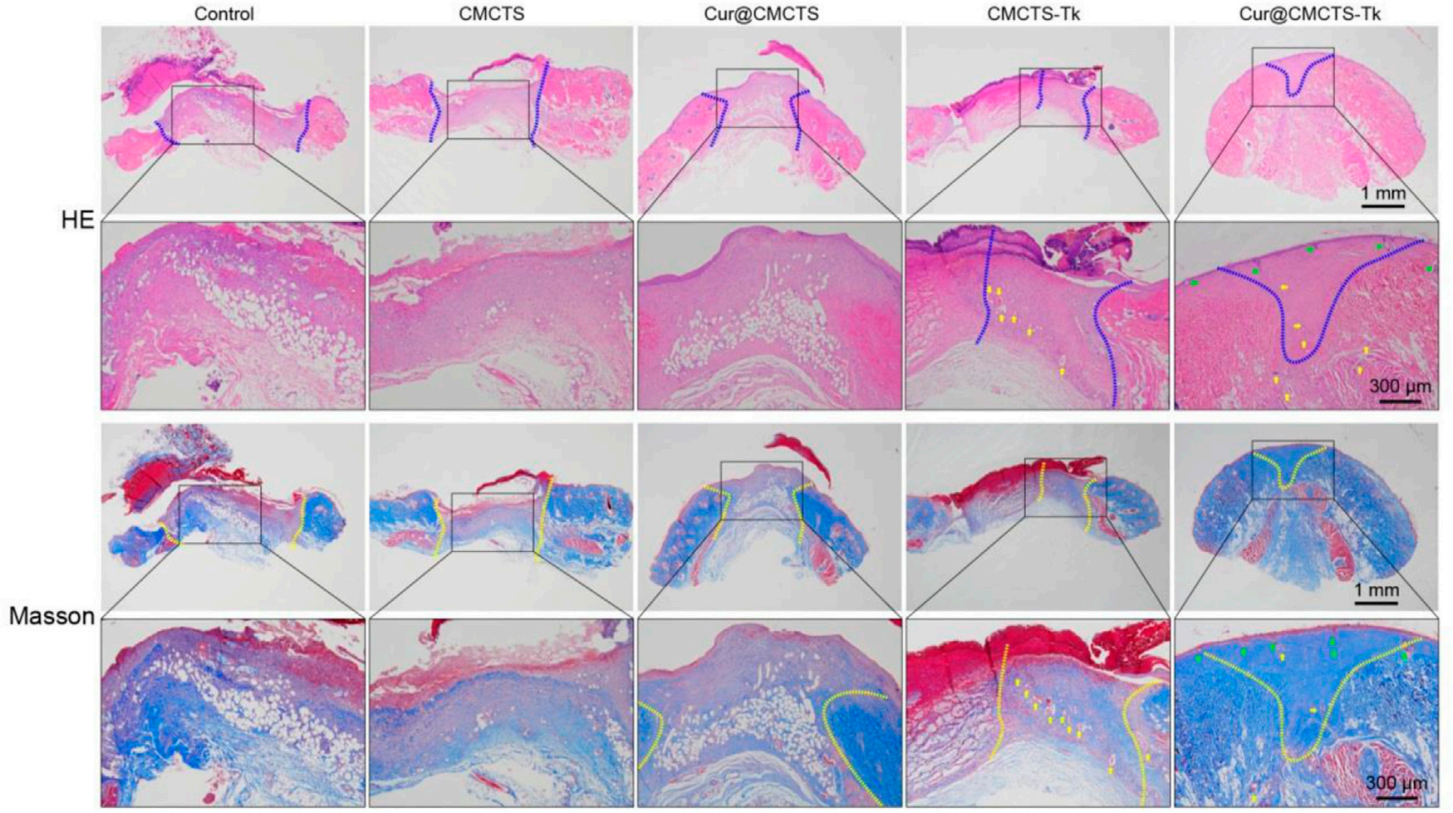
*In vivo* wound healing effect of Cur@CMCTS-Tk hydrogels. Representative images of sections stained with H&E **(top)** and Masson’s trichrome **(bottom)** from normal skin and wounded skin treated with/without hydrogels at day 21 post-wounding (the area within the blue or yellow dashed line is not healed and the yellow arrows represent micrangium). Diminished image scale bars are 1 mm, enlarged image scale bars are 300 µm.

On day 21, we performed histological analysis of the wound using H&E staining and Masson’s trichrome staining (Figure 8). We were surprised to find that the Cur@CMCTS-Tk hydrogel improved reepithelialization and wound remodeling more effectively than the control groups. First of all, the images suggested that the epidermis was similar in thickness to the skin tissue of healthy rats and much thicker than the other groups. These results implied that the burn wound covered by Cur@CMCTS-Tk would obtain limited scar formation due to the ROS elimination ability. In the second place, more blood vessels and hair follicles appeared in the Cur@CMCTS-Tk hydrogel group. Besides, the results of Masson’s trichrome staining showed the Cur@CMCTS-Tk hydrogel group existed higher collagen deposition (blue staining) and more regular fiber arrangement. In the meantime, more microvessels were found in the Cur@CMCTS-Tk group, which was beneficial to wound healing. And this phenomenon can be interpreted as the biological effect of Cur@CMCTS-Tk hydrogel, such as the removal of ROS, continuous release of Cur, inhibition of ROS aggregation, improvement of cell viability, and promotion of angiogenesis. In conclusion, Cur@CMCTS-Tk hydrogel with controllable ROS scavenging activity can accelerate the speed of wound healing and significantly improve the quality of skin tissue regeneration.

### IL-6 and CD31 Expression in the Wound Regeneration Area

**FIGURE 9:**
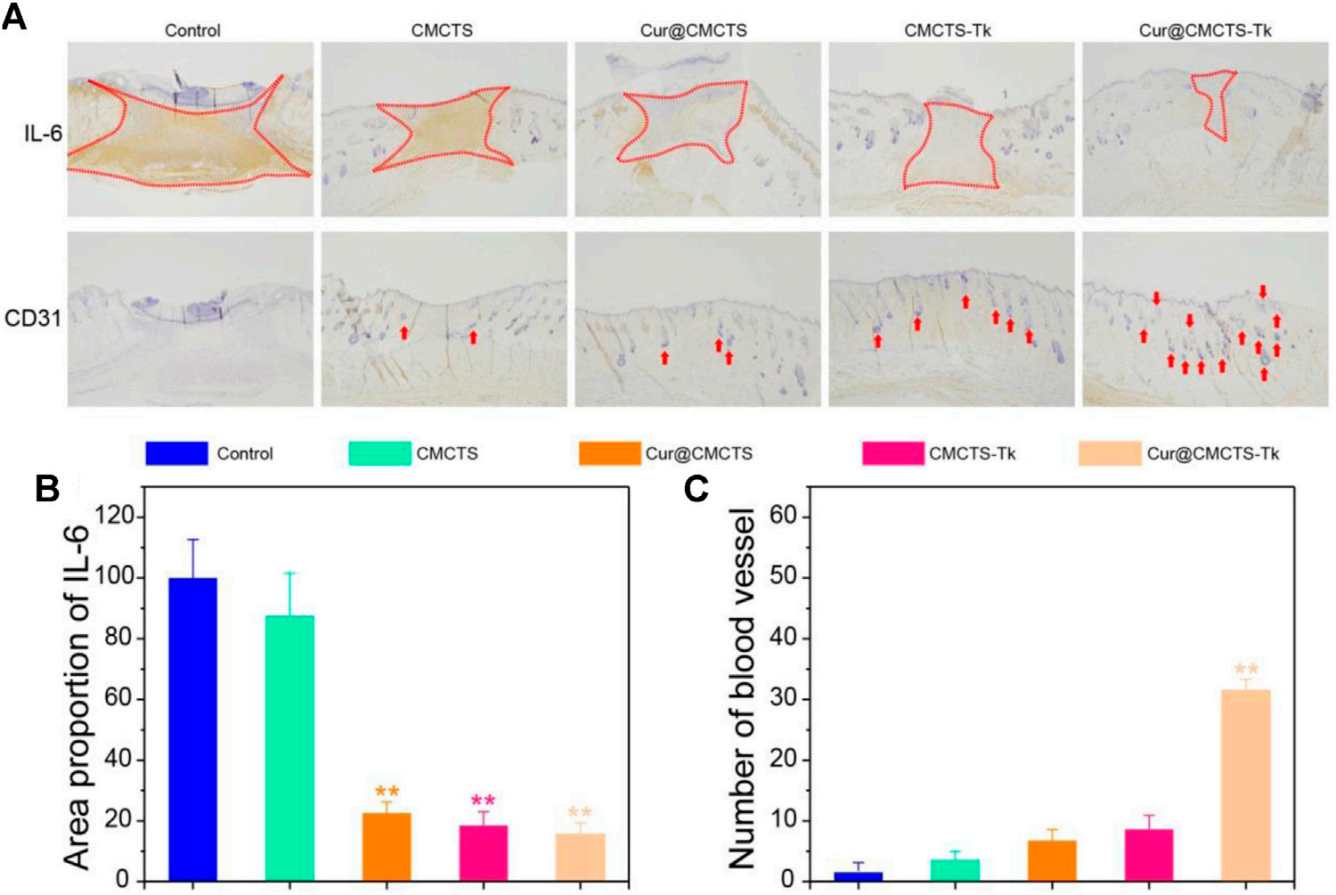
Inflammatory factor and vascularization in the burn wounds treated with Cur@CMCTS-Tk hydrogel. **(A)** IL-6 and CD31 immunohistochemistry staining in the wounds treated with Cur@CMCTS-Tk hydrogel in full-thickness burns rat model at day 21. Expression of IL-6 **(B)** and quantification of the number of blood vessels **(C)** in the wounded area using ImageJ software (*n* = 3).

To verify the inflammation expression in the wound area *in vivo*, we selected IL-6 as the represented cytokine that was closely related to inflammation, so an immunohistochemical method was used to assess the total IL-6 level in the wound area. As Figure 9A shows, the IL-6 expression was higher in the control group, and inflammation was more serious in the CMCTS group on day 21 (**p < 0.01), whereas, the IL-6 expression in CMCTS-Tk and Cur@CMCTS-Tk group was lower in the wound area (Figure 9B), which was due to the efficient ROS scavenging ability of the CMCTS-Tk hydrogel and Cur delivery ability to against inflammatory response. The contents of platelet endothelial cell adhesion molecule-1 (CD31) (DeLisser et al., 1997) that can promote angiogenesis (Figure 9C) in the Cur@CMCTS-Tk group were significantly highest compared to the other groups indicating the fastest angiogenesis and the wound healing rate appeared on day 21 (**p < 0.01). Overall, the intrinsic ROS scavenging ability and Cur delivery capacity of Cur@CMCTS-Tk hydrogel can reduce inflammatory responses and increase angiogenesis to promote wound healing.

## Conclusion

In this study, we developed a Cur@CMCTS-Tk composite hydrogel dressing that progressively delivers Cur to eliminate redundant ROS during inflammation and new tissue formation in the process of wound healing. The Cur@CMCTS-Tk hydrogel, as a continuous phase, could react with ROS and quickly eliminate ROS. With the occurrence of the reaction, the thioketone group broke and the hydrogel degraded, leading to the accelerated release of Cur. Cur@CMCTS-Tk hydrogel presented good water vapor transmittance, mechanical properties, and biocompatibility after H_2_O_2_ treatment and treating with H_2_O_2_ at 37°C could accelerate the delivery of Cur. *In vivo*, Cur@CMCTS-Tk hydrogel could improve the efficiency of wound contraction, reduce the response of inflammation, and promote angiogenesis in the full-thickness burn rat model. Thus, Cur@CMCTS-Tk hydrogel could be a dressing for burn wound treatment.

## Data Availability

The original contributions presented in the study are included in the article/Supplementary Material, and further inquiries can be directed to the corresponding authors.
