# Supplementary material for: ROS-Eliminating Carboxymethyl Chitosan Hydrogel to Enhance Burn Wound-Healing Efficacy
Source: Front Pharmacol. 2021 Jun 14;12:679580. doi: 10.3389/fphar.2021.679580 (PMC8238405; doi:10.3389/fphar.2021.679580)
Supplement: Supplementary file 1 [file Image1.pdf]

ROS-eliminating carboxymethyl chitosan hydrogel to enhance burn wound healing efficacy

Cheng Yang[1, #], Yuhui Chen[1, #], Hai Huang[1], Shicai Fan[1], Chengliang Yang[2], Liping Wang[3], Wenqiang Li[4], Wenxin Niu[5, *], Jianwen Liao[1, *]

[1] Department of Orthopaedic Trauma, Center for Orthopaedic Surgery, The Third Affiliated Hospital of Southern Medical University, Guangzhou 510630, China;

[2] Department of Orthopedics, Affiliated Hospital of Youjiang Medical University for Nationalities, Baise, Guangxi 533000, China;

[3] UniSA Clinical & Health Science, and UniSA Cancer Research Institute, University of South Australia, Adelaide, SA 5001, Australia;

[4] Gungdong provincial engineering technology research center for sports assistive devices, Guangzhou Sport University, Guangzhou 510630, China;

[5] Yangzhi Rehabilitation Hospital, Tongji University School of Medicine, Shanghai 200000, China;

Cheng Yang, and Yuhui Chen: These authors contributed equally to this work.

*Corresponding authors: Wenxin Niu (niu@tongji.edu.cn); Jianwen Liao (JWLiao1213@163.com; 2867705754@qq.com)

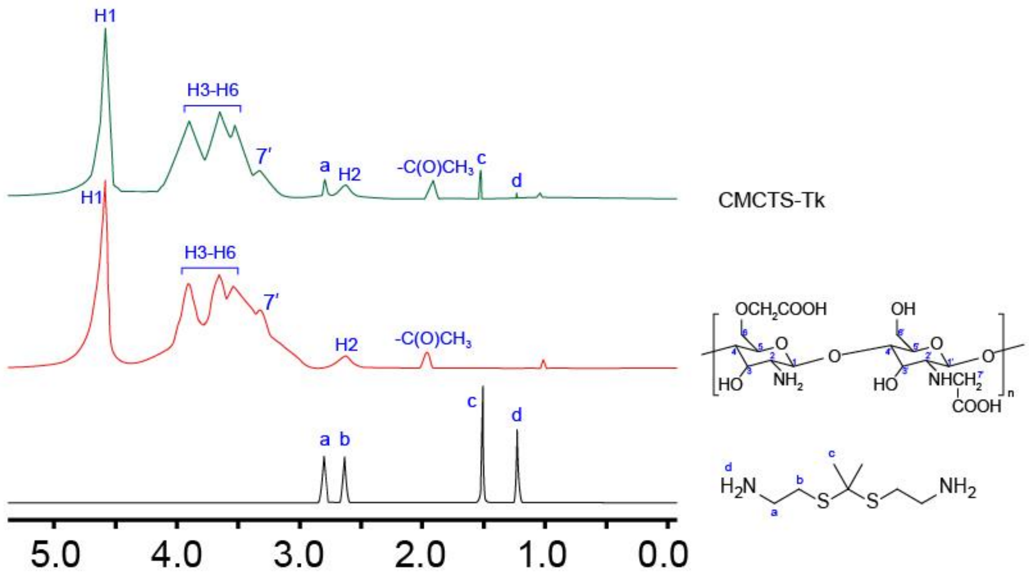

Figure S1. ^{1}H NMR NMR spectra for 2,2'-(propane-2,2-diyldisulfanediyl)diethanamine (Tk), carboxymethyl chitosan (CMCTS) and CMCTS-Tk.
